# Breakfast Food Advertisements in Mediterranean Countries: Products’ Sugar Content in the Adverts from 2015 to 2019

**DOI:** 10.3390/children8010014

**Published:** 2020-12-31

**Authors:** Mireia Montaña Blasco

**Affiliations:** Faculty of Information and Communication Sciences, Universitat Oberta de Catalunya, 08035 Barcelona, Spain; mmontanabl@uoc.edu

**Keywords:** breakfast, childhood obesity, food advertising, food policy, media, Mediterranean diet, sugar-sweetened food

## Abstract

Although Spain was considered to be the healthiest country in the world in 2019, some studies reported that Mediterranean diet (MD) adherence, especially for breakfast, is low among children in Mediterranean countries, where child obesity is increasing alarmingly. This study correlated longitudinally the sugar content of breakfast products with advertising strategies. The research design applied quantitative analysis to compile the advertising data from 2015 to 2019 for all media, qualitative analysis of the content, and the use of popular characters to promote the food purchase. Additionally, a nutritional analysis was used to determine the products’ sugar content. The results were analyzed according to the target they were aimed at (adults or children). Results showed that the Spanish food industry promoted unhealthy products for breakfast, especially those targeted to children, with very high sugar content. To improve the childhood obesity rate in Spain, greater involvement from the food industry is needed. The reformulation of breakfast products must be a priority along with additional sugar reduction strategies so as not to lose adherence to MD in younger generations. More nutrition education is necessary among children, especially on balanced breakfast consumption, a basic meal that helps children to concentrate better in class during the morning.

## 1. Introduction

A complete breakfast that includes fresh ingredients and foods should be a healthy habit for both adults and children, the latter of whom need to concentrate in class during the morning. The regular consumption of an inappropriate breakfast, or even its omission, is associated with many diseases such as obesity, cardiovascular disease (CVD), and diabetes and reduces overall performance during the day. A complete breakfast contains a blend of macronutrients, is rich in protein and fiber, low in sugar and fat, and includes vitamin A, vitamin B, and minerals to minimize fluctuations in insulin and not to predispose to obesity [1,2,3,4].

Although Spain was considered the healthiest country in the world in 2019 [5], some studies reported that adherence to the Mediterranean diet (MD) is low among Mediterranean countries, especially in children and adolescents [6,7]. Previous research highlighted that a global nutrition transition had been fueled by food processing, preservation, and retail [8]. This transition from traditional MD to high-sugar food intake, among many other causes, is increasing the prevalence of obesity and metabolic diseases in Mediterranean countries [9]. The estimated prevalence of overweight in the adult population is about 39% in Spain [10]. It must also be highlighted that about one in three Spanish children is overweight, as stated by the World Health Organization (WHO). The obesity rate among Spanish children has increased critically over the last years, with Spain recently identified as the European country with the highest proportion of childhood obesity [11]. Different studies have shown that low nutritional quality has been observed in the breakfasts that Mediterranean children and adolescents consume. Regarding this fact, the consumption of packaged products that do not require any type of preparation, such as industrial pastries, high-sugar cereal bars, or prepared milkshakes, is increasing [1,12]. Previous research has shown that high-sugared products for breakfast affected adiposity and metabolic parameters. Breakfast products’ nutritional quality is essential for its health benefits [13]. Moreover, regularly feeding children with biscuits or high-sugar products may change their taste preferences and may increase their future consumption of sweeter foods in general [14].

Although there are multiple causes of this dietary pattern transition, advertising can be considered one of them. While some studies have shown that being exposed to repeated persuasive messages also affects adults’ food choices [15], other research obtained opposite conclusions [16]. There is a significant positive association between exposure to child-targeted unhealthy ads and children’s intake of the advertised brands [17]. Different authors have claimed products with low nutritional quality should have their persuasive advertising restricted in order to decrease childhood obesity, as current regulations seem to be ineffective [18,19,20,21].

This research aims to analyze breakfast products’ advertisements, the target they are aimed at, the possible presence of popular characters to encourage their purchase, and the sugar content of the products. To that end, a total of 355 advertisements from 117 different products on Spanish media (internet, television, radio, print, and outdoor) were taken into consideration.

## 2. Materials and Methods

### 2.1. Material Design and Procedure

This study correlated longitudinally the sugar content of breakfast products with advertising discursive strategies. Quantitative analysis was used to compile the advertisements from Infoadex from 2015 to 2019 for all media. This company collects information on Spanish advertising activity, and it has the most complete database of information on the topic. Data are classified in detail: insertions, occupation, and creatives.

We analyzed all the advertisements recompiled by Infoadex. Firstly, we did a qualitative analysis based on all the food advertising campaigns that appealed to breakfast. The word “*desayuno*” (breakfast) had to be in their persuasive discourse. Therefore, a total of 355 advertisements from 117 different products were considered for the content analysis of advertisements: television 39.15%, radio 28.17%, internet 18.03%, press 6.76%, magazines 5.07%, outdoor 2.25%, and cinema 0.56%.

We followed a research protocol to work with these data, and the entire procedure was approved by the Ethical Review Committee of the Universitat Oberta de Catalunya (UOC).

The analysis aimed to answer four research questions: What were the kind of products that were marketed for breakfast time? What was the sugar content of these food products? Do these advertisements use any popular characters to reinforce their persuasive messages? Finally, were there remarkable differences between adults’ and children’s food products and their campaigns? 

### 2.2. Product Category and Advertising Target Analysis

To complete this evaluation, we categorized the advertised products according to the variables “food category” and “sugar component” of each product. The information about the nutritional composition of each product was obtained from the product label to determine the percentage of sugar per 100 g of the product. The author examined all the products to identify those marketed to children or adults. To determine if the ad was targeted to children or adults, the persuasive content was analyzed. Regarding the discourse, ads that had lexical units related to the “childhood” semantic field in their discourse (e.g., kids, school, small, etc.) were considered to be targeted to children. We considered a lexical unit as a word taken in one well-specified sense and supplied with all the information specifying its behavior when it is used in this sense [22]. We understand that a semantic field is a “set of words that share a common core of meaningful characteristics” [23].

To compare the sugar content in the products targeted to adults and those aimed at children, the variation percentage was calculated as (Sugar % in children’s products − Sugar % in Adults products)/Sugar % in children’s products) × 100. The statistical analysis was complemented with the calculation of mean sugar content values. To compare differences in sugar content in adults’ and children’s products, a Mann–Whitney U test was conducted. This non-parametric test was used since the sample did not follow a normal distribution and had non-homogeneous variance. The significance level was set at 5%.

### 2.3. Use of Celebrities or Popular Characters Analysis

We examined the use of celebrities or any kind of character (real, cartoon, or inanimate) that could be known to consumers. A celebrity endorser was defined as any individual who enjoys public recognition and who uses this recognition on behalf of a consumer good by appearing with it in an advertisement [24]. Consequently, four categories of characters were determined: journalists, actors or actresses, athletes, and licensed characters.

## 3. Results

### 3.1. Product Category and Advertising Target Analysis 

The findings indicated that from the 355 breakfast food campaigns between 2015 and 2019, 251 (70.7%) corresponded to an adult target market and 104 (29.3%) were aimed at children (Table 1). Children’s advertisements were those that used any lexical unit from the “childhood” semantic field (e.g., school, kids, break, children, cartoon or animated characters, etc.).

The product category that was most advertised for the adult target market was biscuits (25.10%), followed by cheese (21.12%), coffee (9.96), and yogurts (9.16%). In reference to children, the most advertised product category was biscuits (59.62%), followed by chocolate products (25.00%), and growth milk (6.73%).

While adult products’ advertisements were distributed into 12 food categories, children’s advertisements had less variety, as they were distributed into 7 food categories. 

The nutritional labels of the products of each kind of food were analyzed, and the mean content for each kind of product was determined (Table 2). We observed that a vast majority of products targeted to children had a higher sugar content percentage than the same category of products aimed at adults. The sugar content average was established at 10.25% for the adult products, however, it increased to 36.20% for the products aimed at children. “Biscuits’’ was the category with more adverts for both targets (59.62% of the children’s ads and 25.10% of the adults’ ads), but sugar content varied considerably depending on the product target. The sugar average was 13.34% in the case of adult products and increased to 24.00% for products aimed at children. Another category to highlight is “chocolate products’’ (e.g., chocolate powder and chocolate spread), which represented 25.00% of the children’s advertisements and had an average sugar content of 67.17%. It should be noted that the sugar content in “cereals”, a basic breakfast food, increased 57.3% from products for adults to products for children. A higher standard deviation was found in the “bakery and pastry”, “biscuits”, and “cereals” categories.

To formally test the differences in sugar content in adults’ and children’s products, a Mann–Whitney U test was applied to the total sample of adults’ and children’s products, and the significance level was contrasted. The *z*-score was −11.67147 and the *p*-value was <0.00001 (the result is significant at *p* < 0.05).

### 3.2. Use of Celebrities or Popular Characters Analysis

Regarding the presence of celebrities or popular characters in these campaigns to encourage purchase, we found that this strategy was not commonly used in breakfast product ads (Table 3). In total, 38 popular characters were used in the analyzed adverts. While journalists were the most common characters for the ads targeted to adults, licensed characters (from Star Wars, Pokémon, Mario Kart, Sonic Boom, Invizimals, and Gru 3) appeared in 12 children’s advertisements. This kind of popular character was the only one used in children’s advertising.

The analysis of the nutritional label of the products that each kind of celebrity promoted showed that the products that used athletes for their marketing strategy had an average sugar content of 2.41%, those with journalists in their advertisements had an average sugar content of 7.64%, and the product advertised by an actress had a sugar content of 10.8%. Regarding children’s campaigns, those that used licensed characters, the only well-known “celebrity” used in their case, had an average sugar content of 30.5%.

## 4. Discussion

A traditional Mediterranean breakfast should contain about 30% of the day’s energy and should include whole-grain cereals or bread, milk-based products, and vegetables or fruits. Based on previous research [25,26,27], up to 30% of children and adolescents do not have a qualitatively balanced meal for breakfast or they even skip having breakfast altogether; this trend is especially found in Mediterranean countries, where the rate of obesity in children is higher. Although breakfast intake in these countries seems to be quite balanced for adults [28], children and adolescents have shown poorer adherence to a MD [6], despite the evidence on the association between following a MD and having a healthier life. 

The principal finding from this study was the huge difference between adults’ and children’s breakfast product advertisements, in both the variety and the quality of the products. 

Regarding celebrities and popular characters, the Spanish current regulation [29] indicates that they should not be used in children’s advertisements, based on the influence they could have on the purchase of a product. As this regulation is only applicable to the legal time slot for the protection of minors on television, the recommendations of this code are not effective for the rest of the analyzed media. Children’s discursive strategies for breakfast products claimed the extrinsic and subjective features of the products, as prior studies on general children’s food advertising pointed out [30,31]. Concerning this, it is important to highlight that children do not have developed intellectual abilities to judge persuasive communication from critical reflection. Previous studies have shown that children prefer the innovation of an item or the sense of fun that an advert promotes [32,33]. 

While adults’ advertisements were distributed into 12 food categories, children’s advertisements had less variety, as they were distributed into 7 food categories. Adults’ food categories were also more diverse than the children’s and included no high-sugar cereals, nuts, olive oil, yogurts, or basic MD types of food. The low level of diversity among children’s products reinforces the need for nutritional education among Spanish children to promote a balanced breakfast in order to help prevent obesity. Previous research has shown that obesity is associated with poor food variety consumption and low intake of fruit at breakfast [34]. 

The quality of the products advertised to children was also found to be lower than that of the products advertised to adults. In this sense, children’s cereals contained an alarming percentage of sugar compared to other cereal categories. The improvement of children’s breakfast products needs to be a primary concern in public health strategies to reduce sugar [35]. 

Our recommendations coincide with previous studies that suggested that nutrition education must take into account strategies to promote breakfast intake and to emphasize the importance of having a balanced meal [36]. 

Previous studies have underscored the importance of designing and implementing interventions that address multiple health risk practices, considering lifestyle patterns, such as sedentarism, and associated determinants [37]. As these results showed, public health guidelines might require more emphasis on reducing the intake of added sugars in order to reduce obesity rates [38]. 

Additionally, we suggest the need to establish more effective regulations to restrain food advertisements appealing to children in order to reduce obesity rates. Children’s breakfast product quality, in particular, the amount of added sugars, should be reconsidered by government-set targets and regulations [19]. As a regulatory measure, taxes on high-sugared products might be increased. Previous experience in increasing taxes on sugar-sweetened beverages (SSBs) has shown a remarkable fall in the prevalence of regular consumers of these products, especially among low-income populations [39,40,41]. We must highlight the great increase in ultra-processed foods that almost tripled in Spain between 1990 and 2010 (from 11.0% to 31.7%), paralleling the increase in added sugar content (from 8.4% to 13.0%) [42].

This investigation presents many strengths and some limitations as well. To the best of our knowledge, this is the first study to examine the sugar content of breakfast products and advertising in all media in Spain, the healthiest country in the world in 2019, and one of the most notable representatives of the MD [5]. 

Even though this study analyzes advertising in all forms of media, including the internet, as a limitation, we should contemplate that this medium is very complex and cannot be completely controlled. This study’s focus on one country is another limitation of the research. Nevertheless, the results could represent the Mediterranean countries’ habits. Another limitation of the study is the lack of information about the kind of sugar (e.g., lactose, glucose, etc.) that was used to make the product, as the foods’ packaging did not have this information. Finally, another limitation of this research is the absence of data about people’s breakfast food preferences.

## 5. Conclusions

Although Spain was found to be the healthiest country in the world in 2019, MD adherence among children and adolescents is decreasing significantly in this country, and among all Southern European countries, where childhood obesity is increasing alarmingly. 

Our results seem to indicate that the Spanish food industry promotes unhealthy products for breakfast, especially those targeted to children if we focus on the sugar content, which is about 36.20% on average. From our point of view, public health guidelines might require more emphasis on reducing the intake of added sugars, and the Spanish food industry should be more involved in producing healthier products to reduce obesity rates, especially in childhood. The improvement of children’s breakfast products needs to be a primary concern in public health strategies in order not to lose adherence to MD in young generations. More nutrition education is necessary among children, especially on balanced breakfast consumption, a basic meal that allows children to concentrate at school during morning classes. It should be noted that MD was included on the List of Intangible Cultural Heritage of Humanity by UNESCO (United Nations Educational, Scientific and Cultural Organization) in 2010. Greater efforts must be made to train children in nutritional education, so as not to lose the influence of the MD, which has been so highly appreciated in Spain and the surrounding Mediterranean region.

## Figures and Tables

**Table 1:** Distribution of the adverts by category and consumer target.

Product Category	Ads for Adults Products *n* ^1^ (%)	Ads for Children Products *n ^2^* (%)
Bakery and pastry ^3^	14 (5.58)	1 (0.96)
Biscuits	63 (25.10)	62 (59.62)
Butter	8 (3.19)	N/A
Cereals	18 (7.17)	4 (3.85)
Cheese	53 (21.12)	N/A ^5^
Chocolate products	N/A	26 (25.00)
Coffee	25 (9.96)	N/A
Growth milk	N/A	7 (6.73)
Ham and pâté ^4^	10 (3.98)	1 (0.96)
Jam	17 (6.77)	N/A
Milkshakes	N/A	3 (2.88)
Nuts	6 (2.39)	N/A
Olive oil	3 (1.20)	N/A
Vegetable drinks	11 (4.38)	N/A
Yogurts	23 (9.16)	N/A

^1^ Total *n* = 251; ^2^ Total *n* = 104; ^3^^,4^ In these categories, there is only one ad targeted to children, so the sugar content is related to that specific food. ^5^ No ads for these categories were found.

**Table 2:** Classification of the advertised products by category, average sugar content, and consumer target.

Product Category	Sugar (%) in Adults’ Products ^1^	Sugar (%) in Children’s Products ^2^	Variation ^3^ %
Bakery and pastry ^4^	13.34; *SD* ^6^ = 15.17	24.00	44.4
Biscuits	16.90; *SD* = 6.96	29.23; *SD* = 6.27	42.2
Butter	0.55; *SD* = 0.05	N/A ^7^	N/A
Cereals	10.78; *SD* = 4.48	25.25; *SD* = 0.43	57.3
Cheese	4.01; *SD* = 0.92	N/A	N/A
Chocolate products	N/A	67.17	N/A
Coffee	0.00	N/A	N/A
Growth milk	N/A	5.30	N/A
Ham and pâté ^5^	0.55; *SD* = 0.15	0.40	−37.8
Jam	40.50; *SD* = 0.17	N/A	N/A
Milkshakes	N/A	12.70; *SD* = 0.16	N/A
Nuts	0.00	N/A	N/A
Olive oil	0.00	N/A	N/A
Vegetable drinks	2.64; *SD* = 2.38	N/A	N/A
Yogurts	6.47, *SD* = 3.54	N/A	N/A

^1^ Mean = 10.25; ^2^ Mean = 36.20; ^3^ ((Sugar % in children’s products − Sugar % in Adults products)/Sugar % in children’s products) × 100; Mean= 71.7%; ^4,5^ In these categories, there is only one ad targeted to children, so the sugar content is related to that specific food. ^6^ The standard deviation (SD) is a measure of the amount of variation of a set of values. ^7^ No ads for these categories were found.

**Table 3:** Presence of popular characters in the analyzed adverts.

Presence and Type of Popular Characters	Adults Adverts *n* ^1^ (%)	Children Adverts *n* ^2^ (%)
Non-presence	225 (89.64)	92 (88.46)
Presence	26 (10.36)	12 (11.54)
Journalists	18 (7.17)	N/A ^3^
Actress	1 (0.40)	N/A
Athletes	7 (2.79)	N/A
Licensed characters	N/A	12 (11.54)

^1^ Total *n* = 251; ^2^ Total *n* = 104. ^3^ No ads for these categories were found.

## Data Availability

Data sharing not applicable.
